# Supplementary material for: E69K mutation in β-tubulin 2 blocks cell wall integrity signaling during plant cell elongation
Source: EMBO Rep. 2025 Sep 30;26(21):5117–32. doi: 10.1038/s44319-025-00507-4 (PMC12592460; doi:10.1038/s44319-025-00507-4)
Supplement: Supplementary file 2 — Movie EV1 [file 44319_2025_507_MOESM2_ESM.zip › Movie EV1/Movie EV1.docx]

**Movie EV1. The lateral displacement of cortical microtubules in the *sofa1* mutant.**

Example of the lateral displacement of cortical microtubules labeled by VisGreen-TUB6 in the *sofa1* mutant. Scale bar indicates 2 µm.
