# Supplementary figures and images for: E69K mutation in β-tubulin 2 blocks cell wall integrity signaling during plant cell elongation
Source: EMBO Rep. 2025 Sep 30;26(21):5117–32. doi: 10.1038/s44319-025-00507-4 (PMC12592460; doi:10.1038/s44319-025-00507-4)

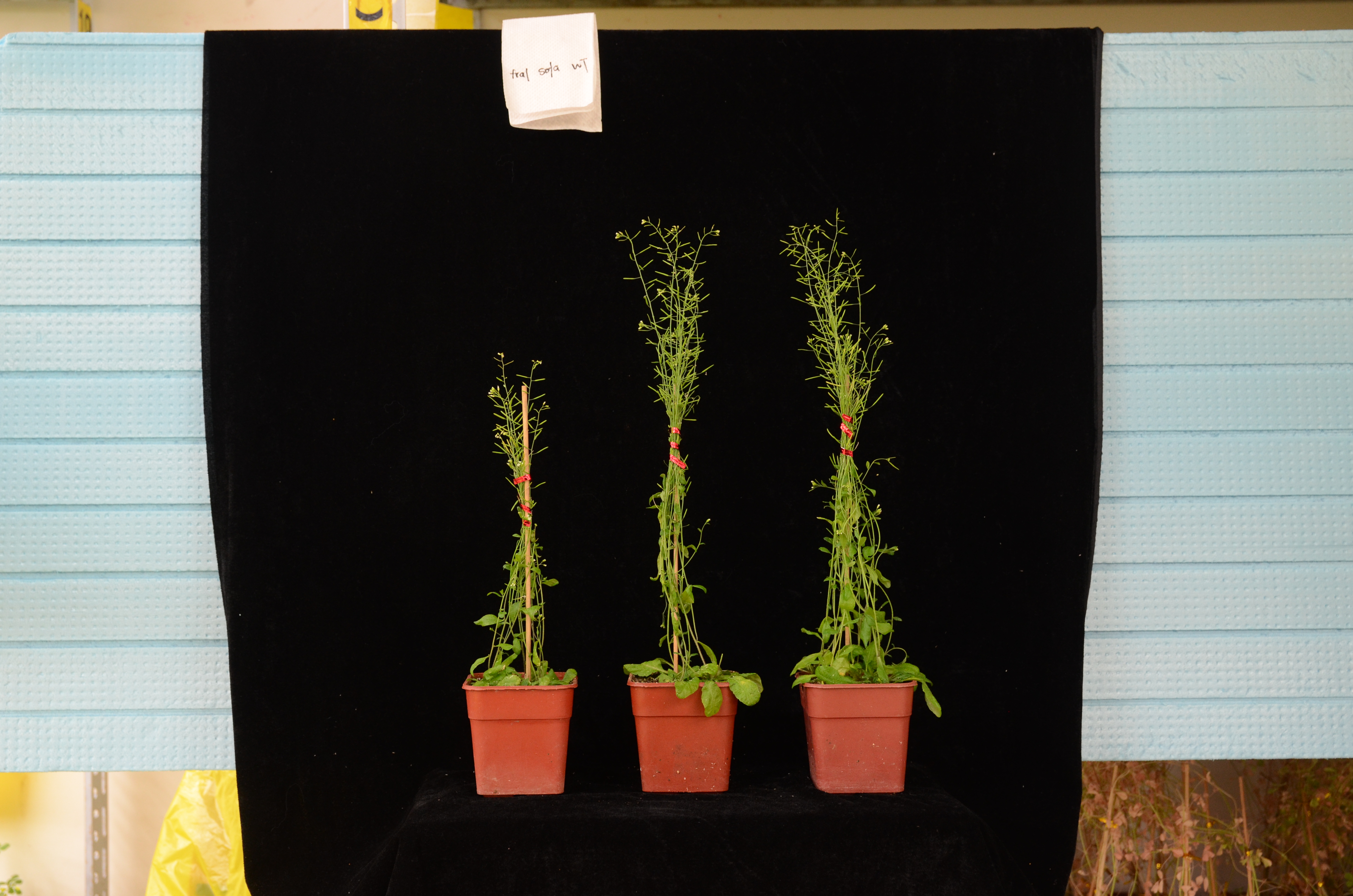

Supplement: Supplementary file 3 — Source data Fig. 1 [file 44319_2025_507_MOESM3_ESM.zip › Figure 1/1A/Growth phenotype.JPG]

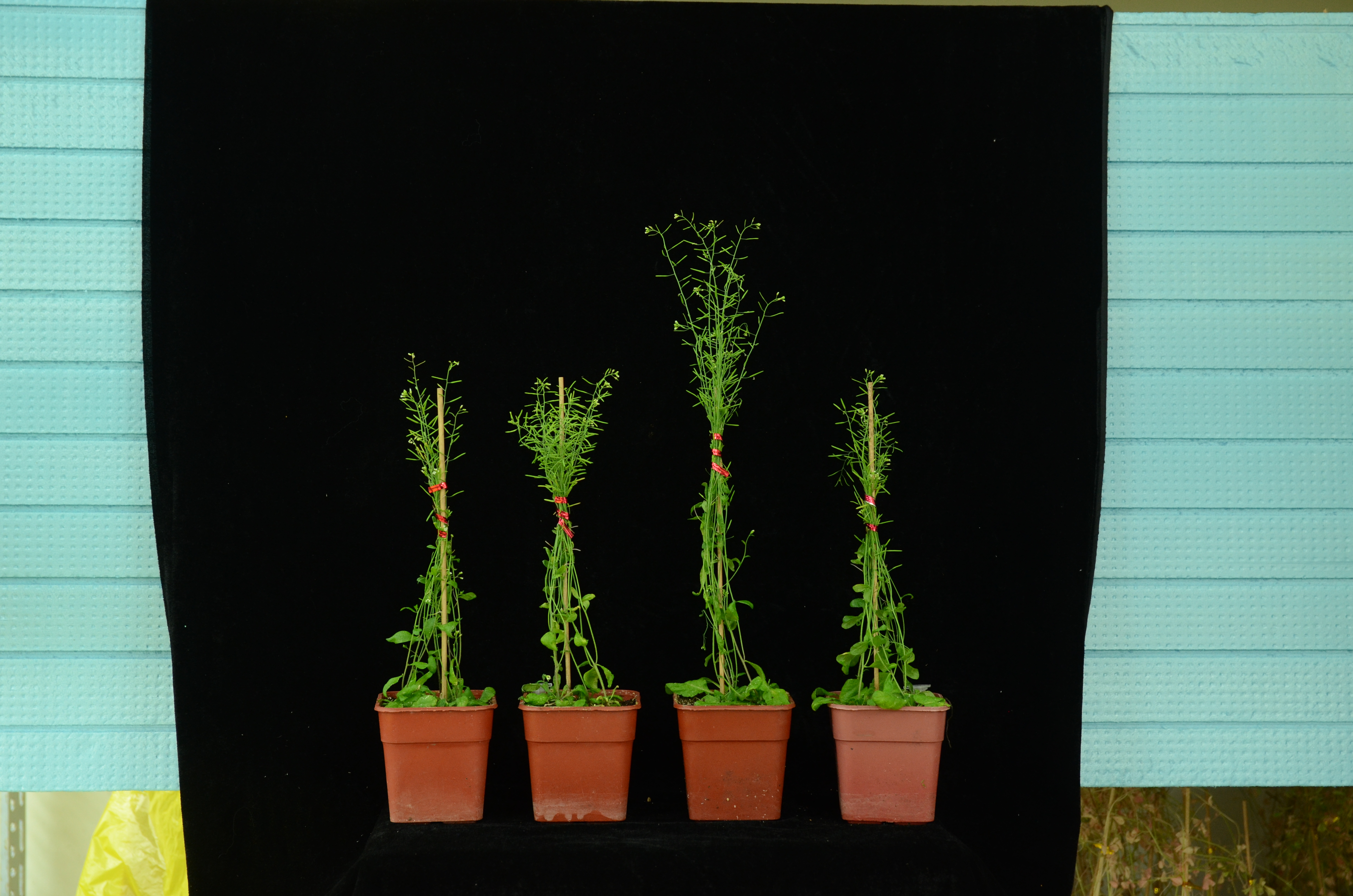

Supplement: Supplementary file 3 — Source data Fig. 1 [file 44319_2025_507_MOESM3_ESM.zip › Figure 1/1G/DSC_4739.JPG]

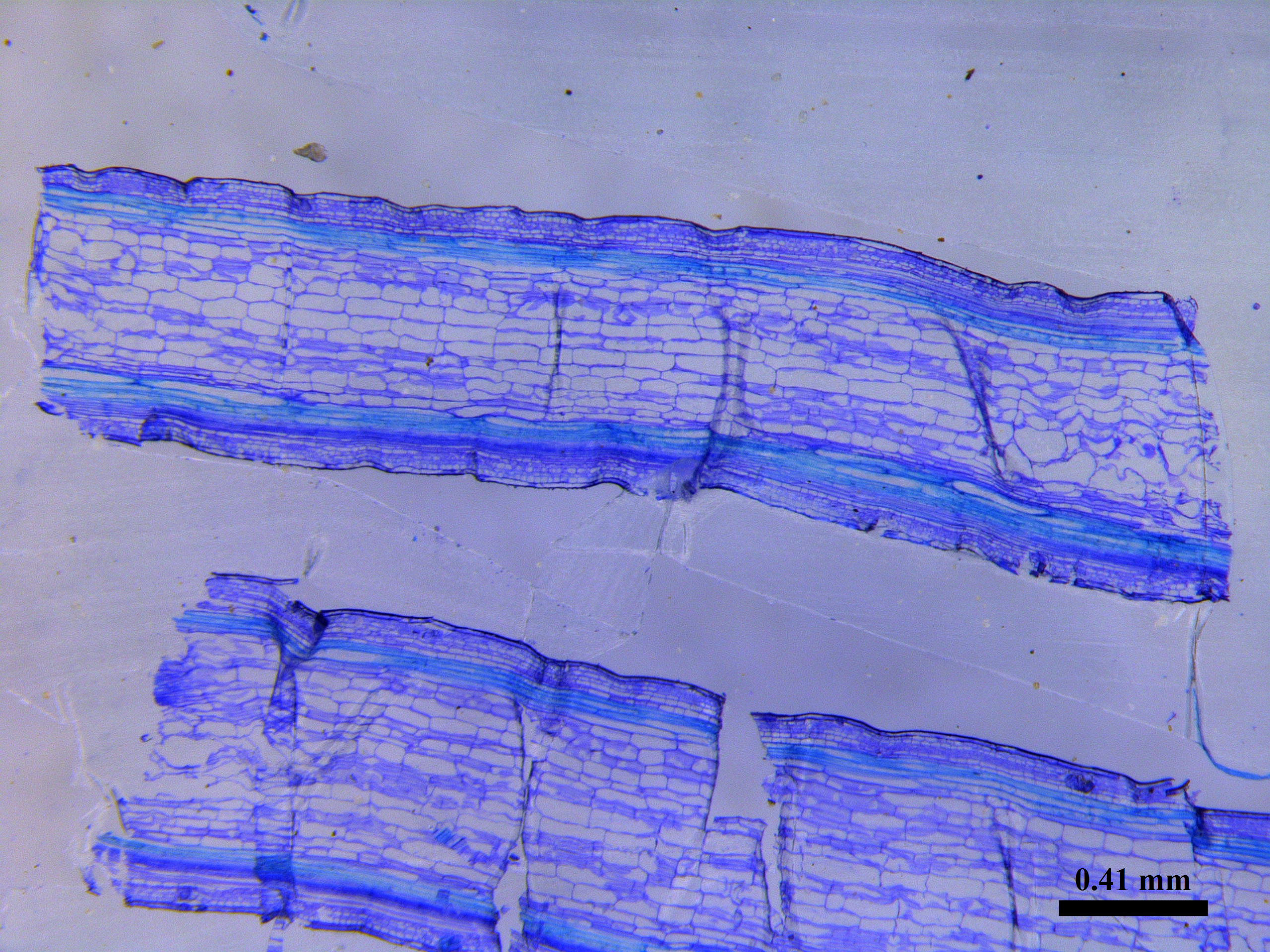

Supplement: Supplementary file 3 — Source data Fig. 1 [file 44319_2025_507_MOESM3_ESM.zip › Figure 1/1C/fra1.tif]

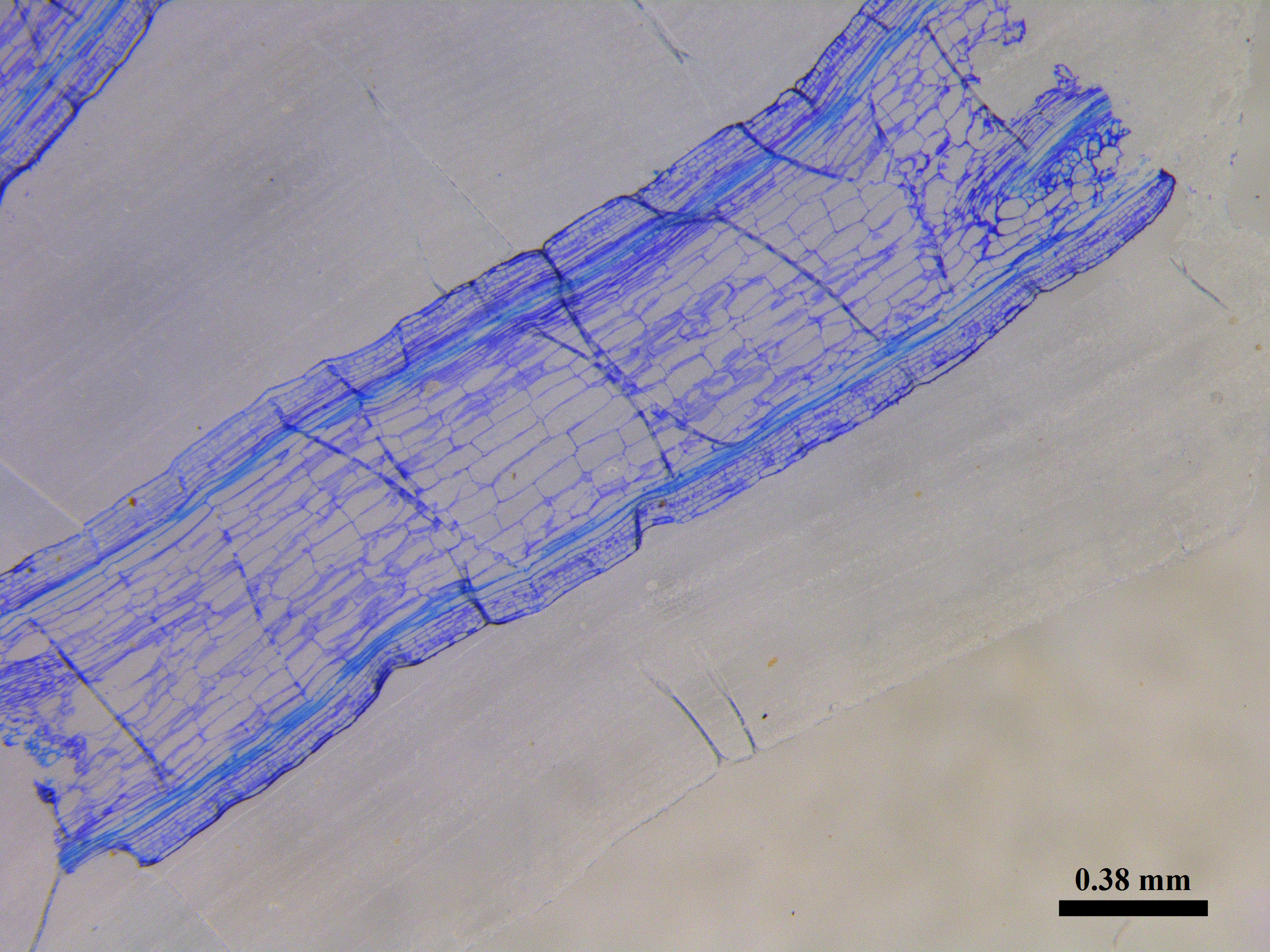

Supplement: Supplementary file 3 — Source data Fig. 1 [file 44319_2025_507_MOESM3_ESM.zip › Figure 1/1C/sofa1.tif]

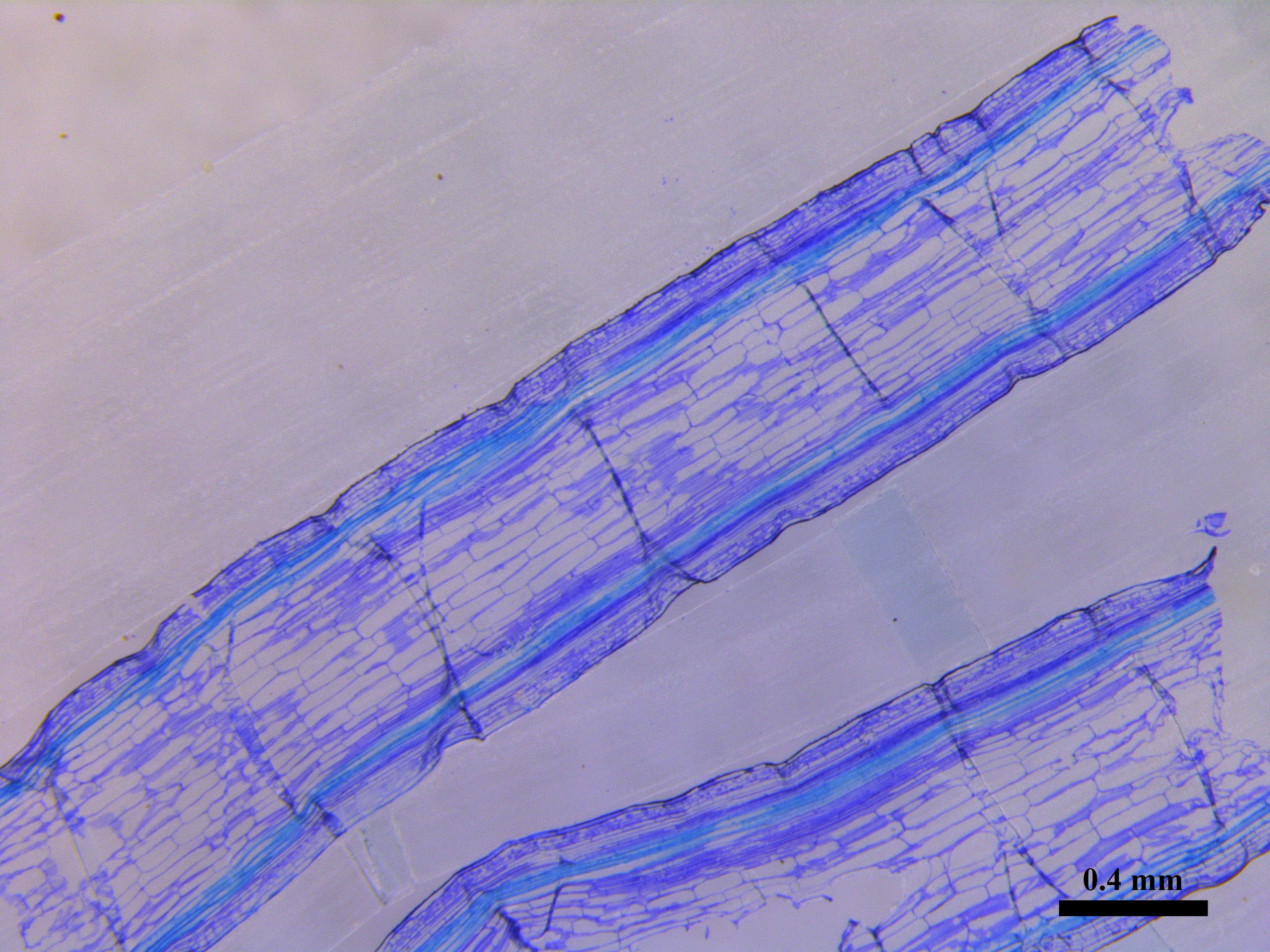

Supplement: Supplementary file 3 — Source data Fig. 1 [file 44319_2025_507_MOESM3_ESM.zip › Figure 1/1C/WT.tif]

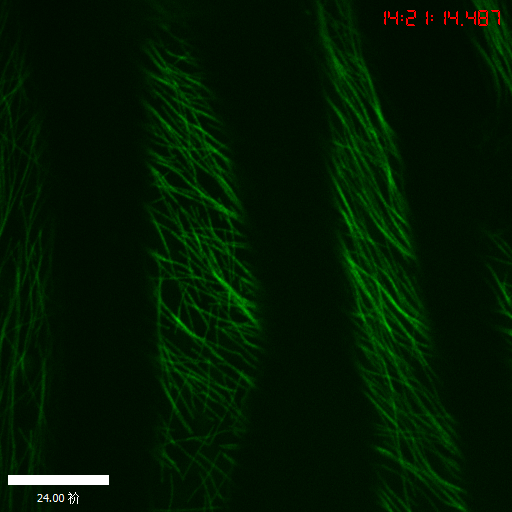

Supplement: Supplementary file 4 — Source data Fig. 2 [file 44319_2025_507_MOESM4_ESM.zip › Figure 2/2G/fra1-tub2-control.tif]

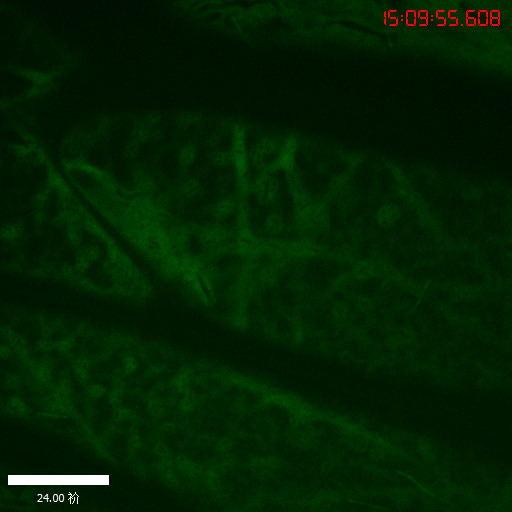

Supplement: Supplementary file 4 — Source data Fig. 2 [file 44319_2025_507_MOESM4_ESM.zip › Figure 2/2G/fra1-tub2-E69K-100uM.tif]

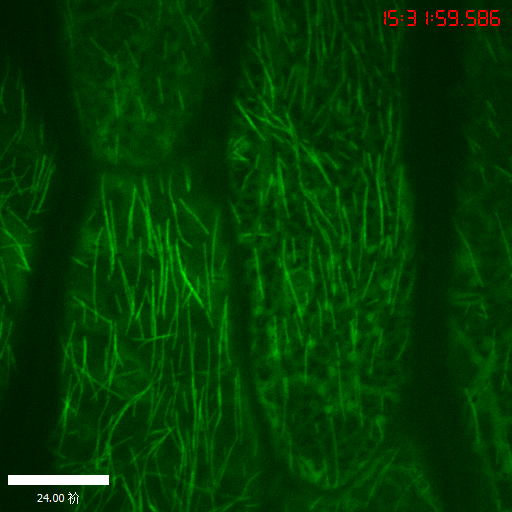

Supplement: Supplementary file 4 — Source data Fig. 2 [file 44319_2025_507_MOESM4_ESM.zip › Figure 2/2G/fra1-tub2-50uM.tif]

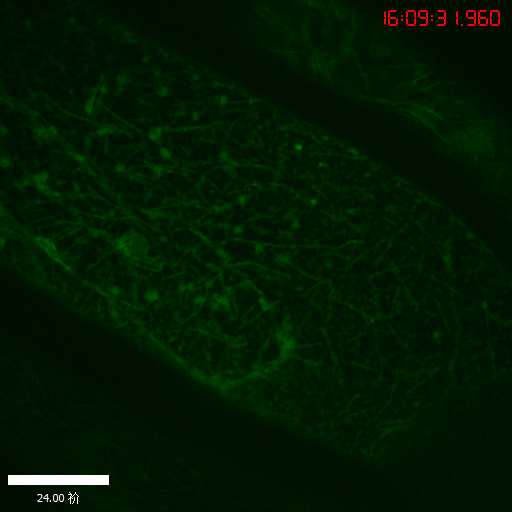

Supplement: Supplementary file 4 — Source data Fig. 2 [file 44319_2025_507_MOESM4_ESM.zip › Figure 2/2G/fra1-tub2-E69K-50uM.tif]

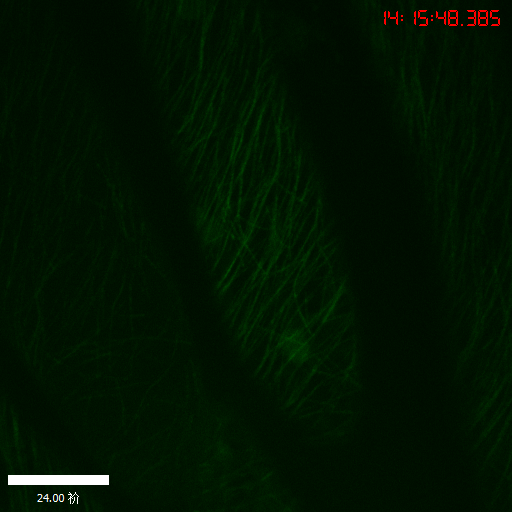

Supplement: Supplementary file 4 — Source data Fig. 2 [file 44319_2025_507_MOESM4_ESM.zip › Figure 2/2G/fra1-tub2-E69K-control.tif]

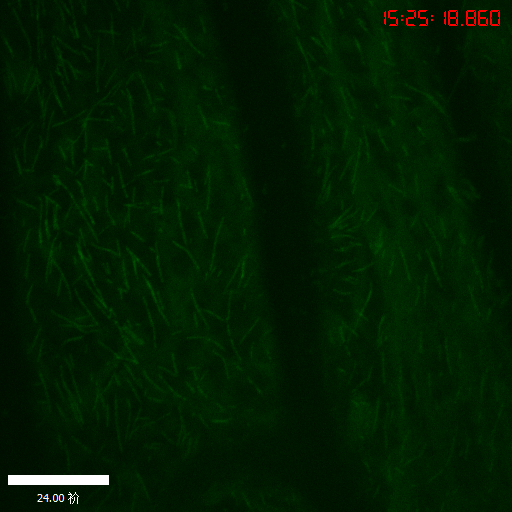

Supplement: Supplementary file 4 — Source data Fig. 2 [file 44319_2025_507_MOESM4_ESM.zip › Figure 2/2G/fra1-tub2-100uM.tif]

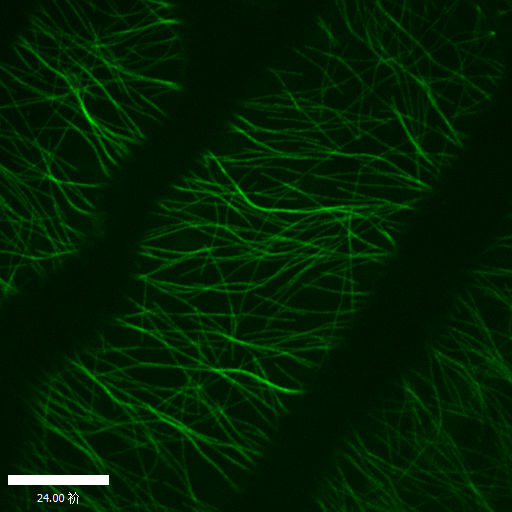

Supplement: Supplementary file 4 — Source data Fig. 2 [file 44319_2025_507_MOESM4_ESM.zip › Figure 2/2A/fra1-tub2.tif]

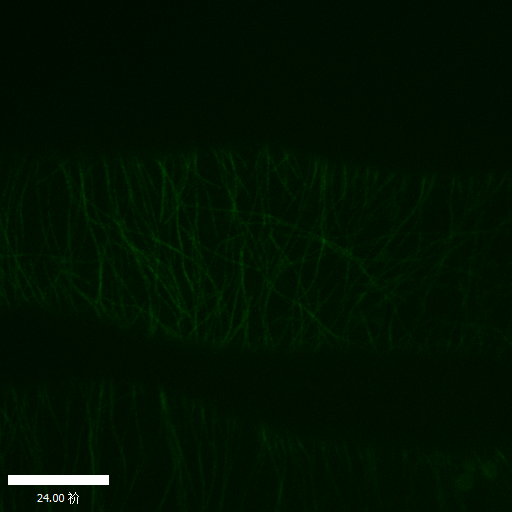

Supplement: Supplementary file 4 — Source data Fig. 2 [file 44319_2025_507_MOESM4_ESM.zip › Figure 2/2A/fra1-tub2-E69K.tif]

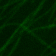

Supplement: Supplementary file 5 — Source data Fig. 3 [file 44319_2025_507_MOESM5_ESM.zip › Figure 3/3B/Magnification .tif]

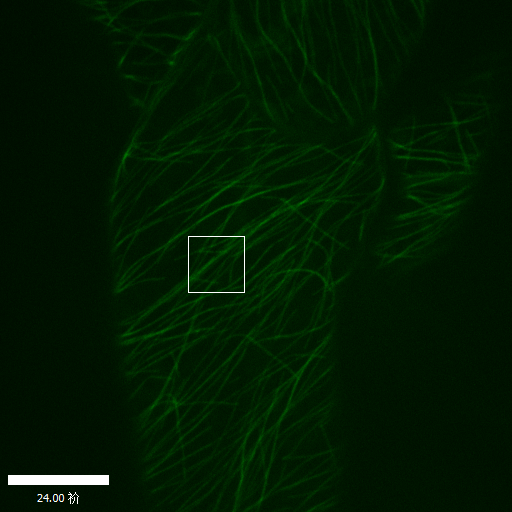

Supplement: Supplementary file 5 — Source data Fig. 3 [file 44319_2025_507_MOESM5_ESM.zip › Figure 3/3B/Original image.tif]

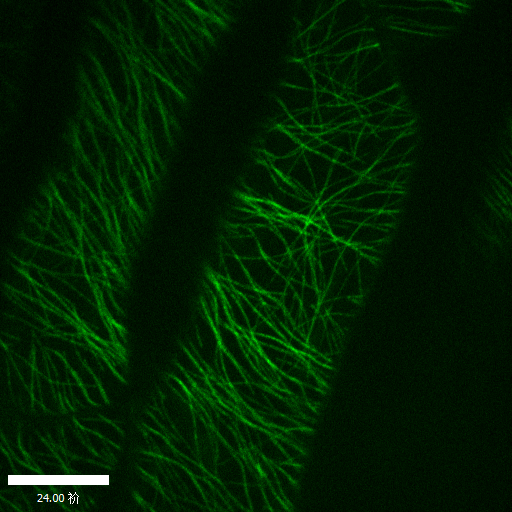

Supplement: Supplementary file 5 — Source data Fig. 3 [file 44319_2025_507_MOESM5_ESM.zip › Figure 3/3C/fra1.tif]

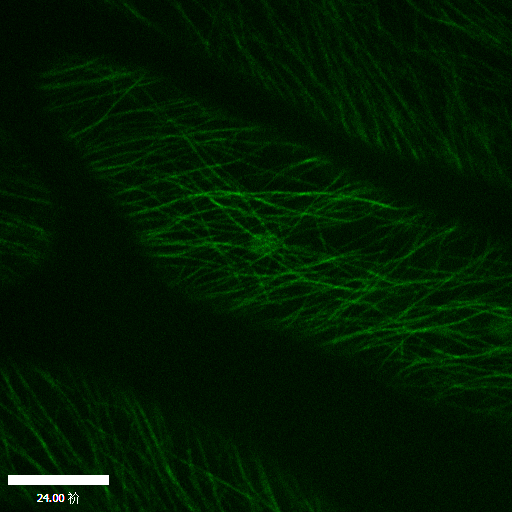

Supplement: Supplementary file 5 — Source data Fig. 3 [file 44319_2025_507_MOESM5_ESM.zip › Figure 3/3C/sofa1.tif]

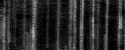

Supplement: Supplementary file 5 — Source data Fig. 3 [file 44319_2025_507_MOESM5_ESM.zip › Figure 3/3C/WT-Kymograph.tif]

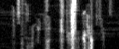

Supplement: Supplementary file 5 — Source data Fig. 3 [file 44319_2025_507_MOESM5_ESM.zip › Figure 3/3C/fra1-Kymograph.tif]

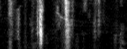

Supplement: Supplementary file 5 — Source data Fig. 3 [file 44319_2025_507_MOESM5_ESM.zip › Figure 3/3C/sofa1-Kymograph.tif]

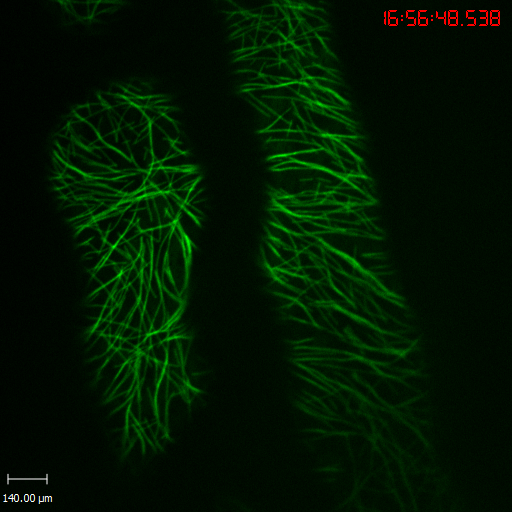

Supplement: Supplementary file 5 — Source data Fig. 3 [file 44319_2025_507_MOESM5_ESM.zip › Figure 3/3C/WT.tif]

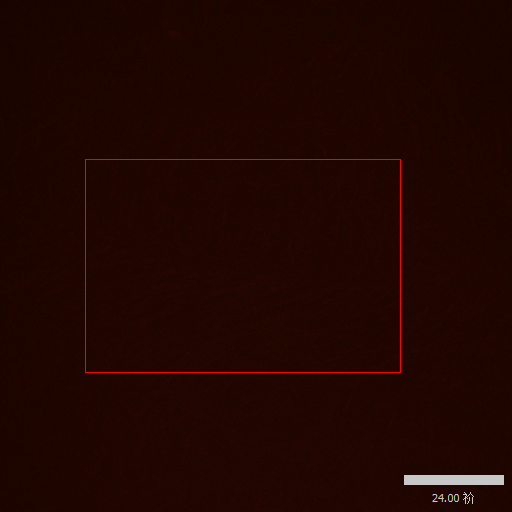

Supplement: Supplementary file 5 — Source data Fig. 3 [file 44319_2025_507_MOESM5_ESM.zip › Figure 3/3A/fra1.tif]

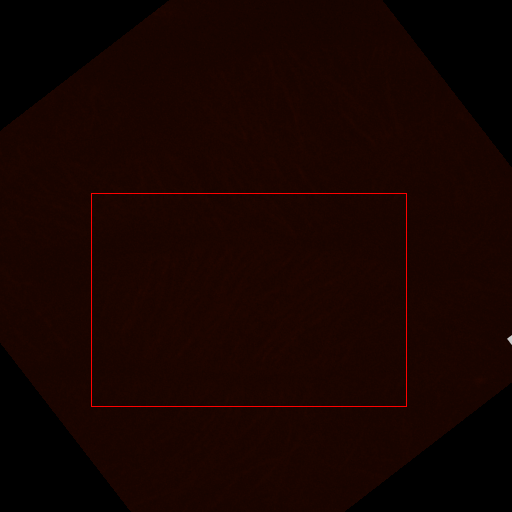

Supplement: Supplementary file 5 — Source data Fig. 3 [file 44319_2025_507_MOESM5_ESM.zip › Figure 3/3A/sofa1.tif]

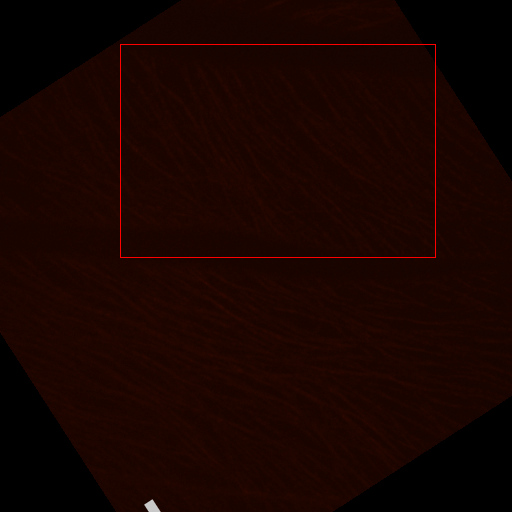

Supplement: Supplementary file 5 — Source data Fig. 3 [file 44319_2025_507_MOESM5_ESM.zip › Figure 3/3A/WT.tif]

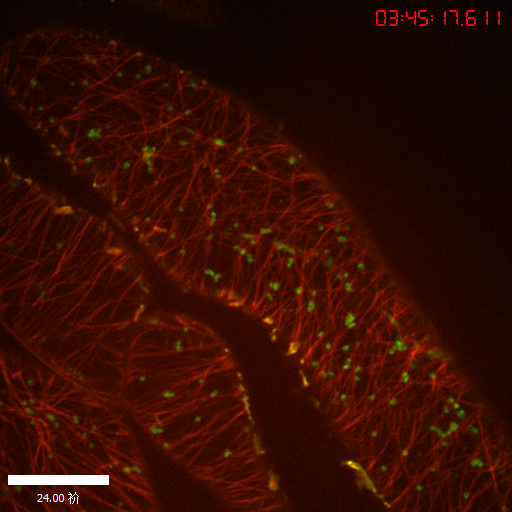

Supplement: Supplementary file 6 — Source data Fig. 4 [file 44319_2025_507_MOESM6_ESM.zip › Figure 4/4D/tub6+cesa3.tif]

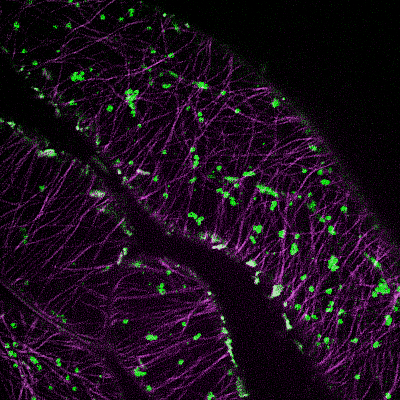

Supplement: Supplementary file 6 — Source data Fig. 4 [file 44319_2025_507_MOESM6_ESM.zip › Figure 4/4D/magenta-GFP.tif]
