# Supplementary material for: E69K mutation in β-tubulin 2 blocks cell wall integrity signaling during plant cell elongation
Source: EMBO Rep. 2025 Sep 30;26(21):5117–32. doi: 10.1038/s44319-025-00507-4 (PMC12592460; doi:10.1038/s44319-025-00507-4)
Supplement: Supplementary file 7 — Expanded View Figures [file 44319_2025_507_MOESM7_ESM.pdf]

Expanded View Figures

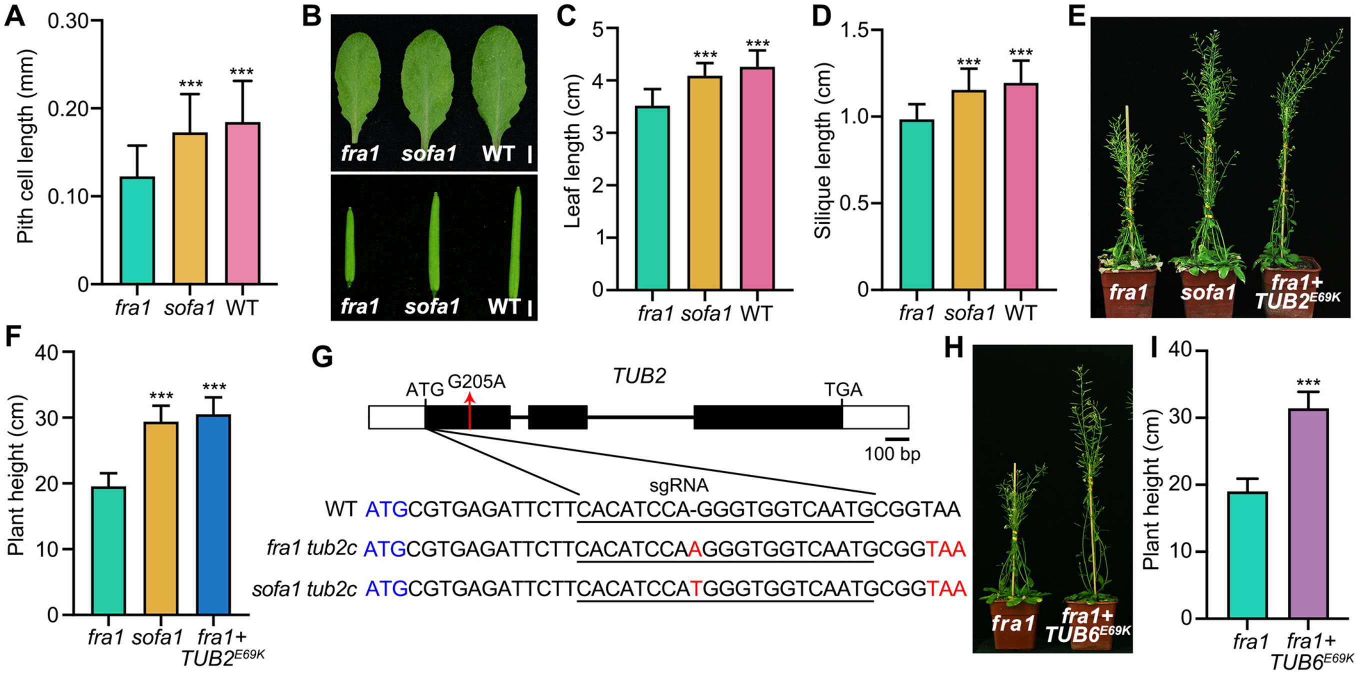

Figure EV1. Identification and gene mapping of the suppressor of *fra1*.

(**A**) Quantification of the pith cell length as shown in Fig. 1C. 50 cells from five individual plants were counted. (**B**) The leaves and siliques of *fra1*, *sofa1* and WT plants. Scale bars indicate 5 cm (upper) and 2 cm (lower), respectively. (**C**, **D**) Quantification of the length of leaves (**C**) and siliques (**D**) of *fra1*, *sofa1* and WT plants as shown in (B). 20 leaves and 50 siliques from five individual plants were counted. (**E**, **F**) Growth phenotype of *fra1*, *sofa1* and *fra1* + $TUB2^{E69K}$ plants (**E**) and quantification of their plant height (**F**). More than 20 plants were counted for each sample. (**G**) A schema of the *TUB2* gene showing the CRISPR/Cas9 targeted site in different mutant lines. The single guide RNA (sgRNA) site is indicated by underlines. The start codons (ATG) are shown in blue letters; base insertion (A/T) and premature stop codons (TAA) are denoted with red letters. (**H**, **I**) Growth phenotype of *fra1*, *fra1* + $TUB6^{E69K}$ plants (**H**) and quantification of their plant height (**I**). More than 20 plants were counted for each sample. Data information: In (**A**, **C**, **D**, **F**), data are presented as mean ± SD. ***$P < 0.001$ compared with *fra1* by one-way ANOVA. In (**A**), $P < 0.0001$ (*sofa1*), $P < 0.0001$ (WT). In (**C**), $P = 0.0005$ (*sofa1*), $P < 0.0001$ (WT). In (**D**), $P < 0.0001$ (*sofa1*), $P < 0.0001$ (WT). In (**F**), $P < 0.0001$ (*sofa1*), $P < 0.0001$ (*fra1* + $TUB2^{E69K}$). In (**I**), data are presented as mean ± SD. ***$P < 0.001$ compared with *fra1* by Student's *t* test, $P < 0.0001$.

© The Author(s)

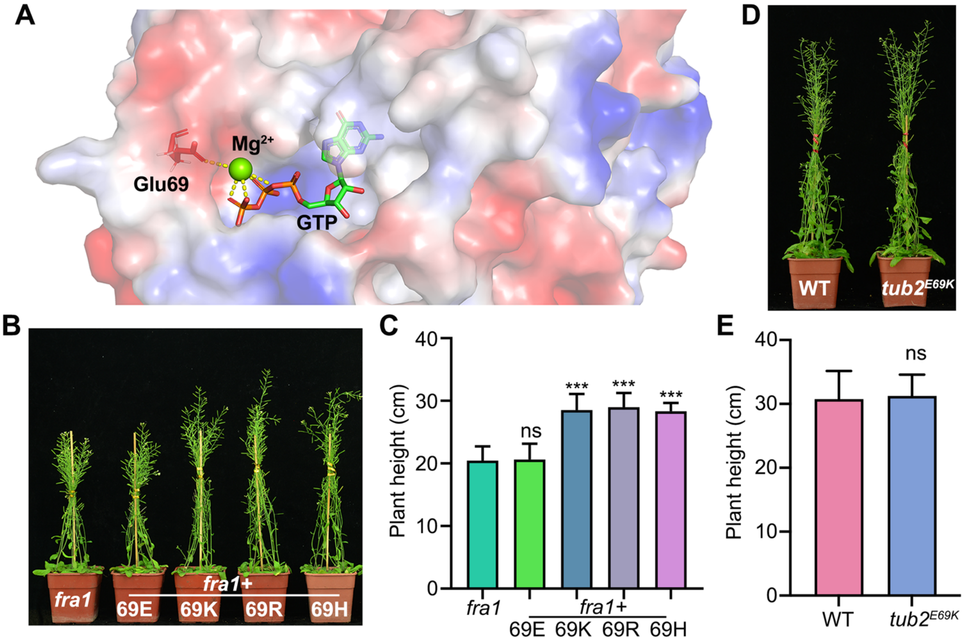

Figure EV2. The 69th residue of TUB2 plays a key role in plant height control of *fra1*.

(**A**) The resolved crystal structure of yeast β-tubulin (PDB:4FFB), accommodating a Mg^{2+} ion (green sphere) and a GTP (sticks colored by atom type). The 69Glu (red stick) is highlighted. (**B**, **C**) Growth phenotype of *fra1*, *fra1* + $TUB2^{69E/K/R/H}$ (*fra1* + 69E, 69K, 69 R, 69H) plants (**B**) and quantification of their plant height (**C**). More than 20 plants were counted for each sample. (**D**, **E**) Growth phenotype of WT and $tub2^{E69K}$ plants (**D**) and quantification of their plant height (**E**). More than 20 plants were counted for each sample. Data information: In (**C**), data are presented as mean ± SD. ***$P < 0.001$ and ns ($P > 0.05$), no significant difference with *fra1* by one-way ANOVA, $P = 0.9974$ (69E), $P < 0.0001$ (69K), $P < 0.0001$ (69 R), $P < 0.0001$ (69H). In (**E**), data are presented as mean ± SD. ns ($P > 0.05$), no significant difference with WT by Student's *t* test, $P = 0.1026$.

© The Author(s)

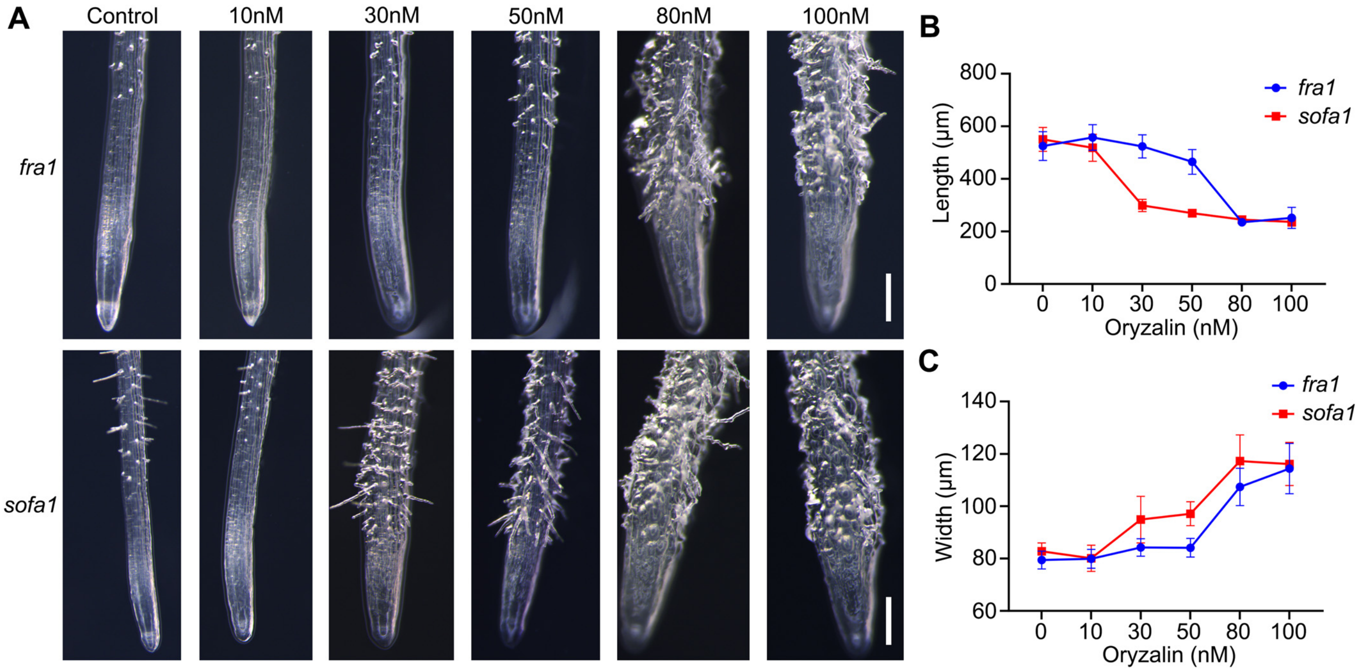

Figure EV3. The incorporation of TUB2^{E69K} influences the stability of microtubules.

(**A**) Primary roots of 6-day-old *fra1* and *sofa1* seedlings exposed for 24 h to various concentrations of oryzalin. Scale bars indicate 100 µm. (**B**, **C**) Quantification of the root length (**B**) and root width (**C**) as shown in (**A**). Data are presented as mean ± SD. $n = 8$ biological replicates.

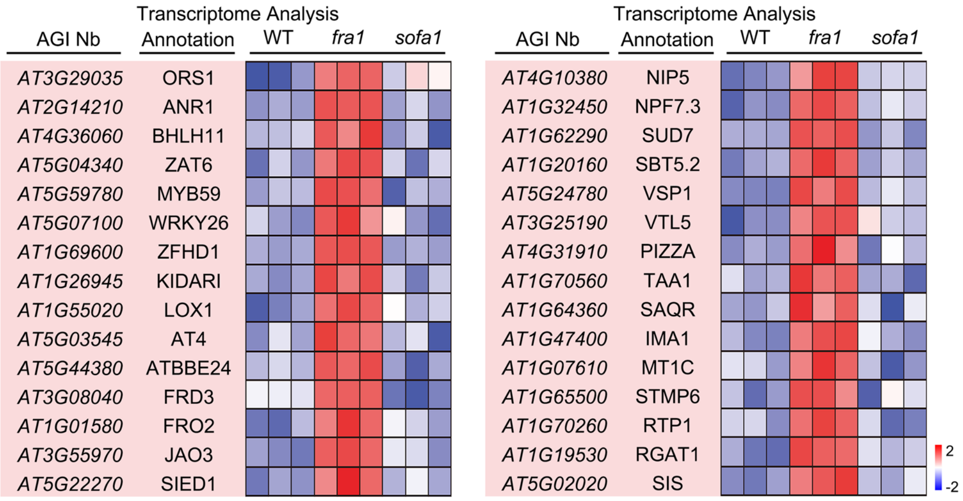

Figure EV4. The expression levels of stress-related genes in WT, *fra1* and *sofa1*.

The heatmap showing the expression levels of a total of 30 stress-related genes in WT, *fra1* and *sofa1* inflorescence stems. Colored bar represents z-score of log2-transformed relative expression.

 © The Author(s)
